# Pharmacists in OPAT: a systematic review and meta-analysis

**DOI:** 10.1093/jac/dkag237

**Published:** 2026-07-15

**Authors:** Yasmin Elmasry, Kezia Wong, Sarah Jones, Anneka Mitchell, Matthew Jones

**Affiliations:** Department of Life Sciences, University of Bath, Bath, UK; Pharmacy Department, University Hospitals Plymouth NHS Trust, Plymouth, UK; Department of Life Sciences, University of Bath, Bath, UK; Pharmacy Department, WestHertfordshire Teaching Hospitals NHS Trust, Hertfordshire, UK; Department of Life Sciences, University of Bath, Bath, UK; Department of Life Sciences, University of Bath, Bath, UK; Pharmacy Department, University Hospitals Plymouth NHS Trust, Plymouth, UK; Department of Life Sciences, University of Bath, Bath, UK

## Abstract

**Background and objectives:**

Outpatient parenteral antimicrobial therapy (OPAT) is defined as the administration of at least two doses of parenteral antimicrobials in an outpatient setting without an overnight hospital admission. OPAT has demonstrated positive patient outcomes whilst reducing bed pressures in hospital and exposure to healthcare-associated infections. International infection societies stress the importance of a core multi-disciplinary team to manage OPAT services. However, many services do not have a pharmacist as part of this core group, with few studies evaluating their effectiveness. To evaluate current evidence for the impact of pharmacists working in OPAT settings on patient safety and outcomes.

**Methods:**

A PRISMA protocol was developed and published (PROSPERO CRD42023452470). Database literature search completed from conception to April 2025. Studies were required to have a pharmacist as part of the multi-disciplinary team alongside a comparator cohort.

**Results:**

We identified 918 studies, with ten meeting the eligibility criteria and all demonstrating a high risk of bias. Pharmacist intervention was found to increase adherence to therapeutic drug monitoring and antibiotic dose adjustment by 40–64% and reduce antimicrobial prescribing errors by 17%–22%. Complications, including re-admissions; emergency department (ED) visits and unfavourable outcomes, had less consistent results across studies.

**Conclusion:**

The findings of this systematic review and meta-analysis indicate that the involvement of pharmacists was associated with improvements in process outcomes such as adherence to drug monitoring protocols, but associations with health outcomes were less consistent. Further studies with robust protocols are required.

## Introduction

Outpatient parenteral antimicrobial therapy (OPAT) is a rapidly developing area with unique antimicrobial stewardship (AMS) considerations. OPAT is defined as the administration of at least two doses of parenteral antimicrobials in an outpatient setting without an overnight hospital admission.^1^ It has been used to facilitate early discharge of patients, or in some cases, avoid admission to hospital altogether.

OPAT is increasingly recognized as a key component of AMS programmes, supporting appropriate antimicrobial selection, earlier intravenous-to-oral switch where appropriate, reduced hospital exposure, thereby lowering the risk of healthcare-associated infections and contributing to improved patient outcomes, including quality of life.^2–5^ In addition, the COVID-19 pandemic created an opportunity to expand the scope and capabilities of OPAT services. Although OPAT has been well established for many years, with early use in cystic fibrosis patients,^6^ the pandemic necessitated rapid adaptation and facilitated service evolution in response to a changing healthcare landscape.^7^

The BSAC OPAT good practice recommendations stress the importance of implementing a formal OPAT service structure with studies including a multi-disciplinary infectious disease team demonstrating a positive impact on patient safety, cost-effectiveness and regimen simplification.^8^ The recommendations describe clinical antimicrobial pharmacists as core staff required for the successful operation of an OPAT service and hence advise that they form part of the team. These recommendations have been supported by good practice recommendations made by other countries such as the Republic of Ireland,^9^ USA^10^ and Australia.^11^

The recent introduction of virtual wards in the UK has created further opportunities for OPAT services. Virtual wards are a hospital-at-home model of care in which patients who would otherwise require inpatient admission receive active clinical monitoring and treatment in their own home under multi-disciplinary supervision.^12^ This model facilitates hospital-level care in the community, including administration of intravenous antimicrobials and regular nursing review, thereby supporting earlier discharge and helping to reduce inpatient bed pressures.^12^ Within the National Health Service (NHS) 10-year plan, virtual wards are positioned as a key mechanism for delivering acute care in the community; however, they are not intended to function as standalone OPAT services. Updated guidance published in April 2025 further supports the development and expansion of OPAT services, including opportunities for alignment with virtual ward models, with the aim of improving patient outcomes and strengthening AMS practices.^13^

Variations in staffing levels across UK healthcare settings mean that at least one-third of OPAT services registered with the BSAC OPAT service directory do not report including a pharmacist within their multi-disciplinary team; however, as completion of this field is optional, this may not accurately reflect true staffing levels.^14^ Comparable national directories are not clearly established in other countries that utilize OPAT services, such as the USA, limiting the ability to draw direct international comparisons in healthcare staffing. Similarly, in Australia, antimicrobial utilization data are collected through the National Antimicrobial Utilisation Surveillance Programme with pharmacists contributing to the data collection, but there is limited insight into how OPAT team structures are organized.^11^

While the role of clinical antimicrobial pharmacists has evolved, there remains limited synthesis of evidence evaluating the impact of pharmacist-led or pharmacist-involved interventions on patient safety and outcomes within OPAT services, despite their established role in AMS governance and service delivery and their recommended role as a core member of the OPAT multi-disciplinary team.

## Objective

The objective of this systematic review was therefore to evaluate current evidence for the impact of pharmacists working in OPAT settings on patient safety and outcomes.

## Methods

The Preferred Reporting Items for Systematic Review and Meta-Analysis (PRISMA) was used for this systematic review. The protocol was published in advance (PROSPERO CRD42023452470).^15^

### Eligibility criteria

For this systematic review and meta-analysis, we searched without data or language restriction for all primary peer-reviewed quantitative studies. Studies with participants of any age who received OPAT were included; studies were required to have a pharmacist as part of the multi-disciplinary team with clear details of interventions made, and a comparator cohort. All studies without a comparator group (either a baseline/pre-implementation or control group) were excluded. Studies had to include outcomes relating to the safety or effectiveness of the intervention. Studies with a focus on a specific antibiotic without any intervention were excluded. Conference abstracts were excluded.

### Search strategy

The search strategy was developed by Y.E. with assistance from University of Bath librarians. Search terms were developed with synonyms for outpatient, parenteral, antibiotics, pharmacist, pharmacy and OPAT. Terms related to the outcome were not specified to maximize the number of studies included. The full search strategy is included in Appendix S1 (available as Supplementary data at *JAC* Online).

We searched PubMed, ProQuest, Embase, Web of Science, CINAHL and Cochrane library databases from date of database conception to April 2025. All non-English studies were translated using Google Translate.

### Study selection

Duplicate results were removed, and Y.E. screened all abstracts against the study protocol using Rayyan AI software and Endnote.^16,17^ Full-text screening was completed by K.W. and Y.E. independently. Disagreements were resolved through consensus or discussion with a third party (M.J.).

### Assessment of quality

The Newcastle-Ottawa Score (NOS) was used to assess the risk of bias of case–control and cohort observational studies.^18^ Studies were given a score from 0 to 9, with studies rated 0–2 being considered poor quality and at very high risk of bias, 3–5 were fair quality and 6–9 were considered high quality with the lowest risk of bias. The scoring was completed independently by three authors (A.M., M.J. and Y.E.), disagreements were then resolved through discussion. Studies were not excluded based on their quality.

### Data extraction

Data were extracted by one reviewer (Y.E.) following the Cochrane checklist for data collection considerations^19^ into an Excel spreadsheet. The following data were extracted from the included studies: author, year, title, country and study type, type of OPAT setting, sample size and patient characteristics. In addition, the inclusion and exclusion criteria, type of infections and antibiotics used during OPAT, pharmacist intervention details, and primary and secondary outcomes (if any) were recorded. No attempts were made to contact the authors to clarify published data.

### Data synthesis and meta-analysis

The data from each study were grouped by type of pharmacist intervention and pre-specified study outcome groups, including clinical effectiveness, cost-effectiveness, quality of care and complications (adverse effects, re-admissions, infection relapse and mortality).

Where possible, using the published data, odds ratios comparing the intervention and comparator group were calculated and meta-analysis was performed using Stata v18.^20^ Heterogeneity was assessed using the *I*^2^ statistic^21^ and meta-analyses were conducted using fixed inverse variance effect^22^ and random effect^23^ models for comparison. Where meta-analysis was not appropriate, studies were summarized using descriptive statistics.

## Results

### Study selection and characteristics

**Figure 1.**
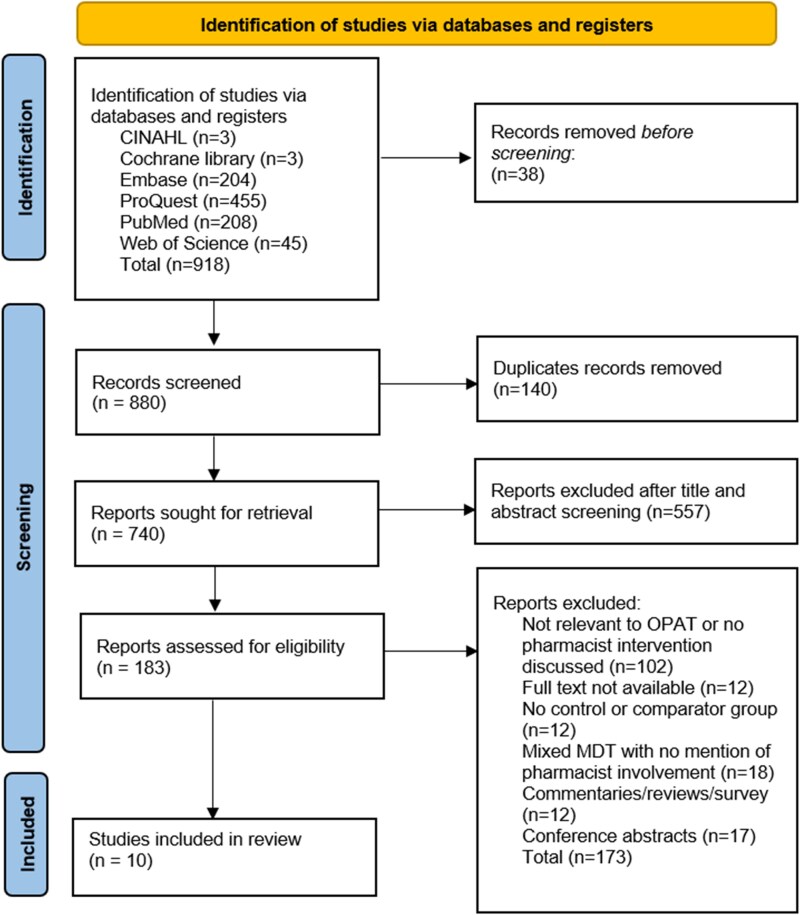
PRISMA flow diagram.

Ten studies met the inclusion criteria and were included for analysis (Figure 1). All were observational cohort studies, with two studies including a historical control group,^24,25^ six studies had a pre-intervention and intervention group^26–31^ and two studies had concurrent intervention and control groups.^32,33^ All studies were conducted in the United States, except for one in Thailand^24^ and one in Belgium.^31^

Nine studies provided OPAT services in one specific location, five were in patients’ homes,^26,27,30,31,33^ two were conducted in infusion centres,^24,25^ one in a community hospital^32^ and one in an OPAT clinic.^29^ One of the studies provided OPAT services in multiple locations including infusion centres, urgent care centres, ED or patient’s homes.^28^ Only one study focused on paediatric OPAT^27^ while the rest studied adults.

Three studies did not state participants’ gender,^27,29,32^ although Hemenway *et al.*^29^ stated that the age and gender distribution were similar across both cohorts.^29^ With the exception of Wong *et al.*^28^ who focused on the management of uncomplicated urinary tract infections caused by ESBL-producing Enterobacteriaceae, all studies included varying infections and antimicrobial agents. Three studies did not state the antimicrobial agents used in their studies.^24,27,29^

Four studies focused on pharmacist-led interventions.^24,25,32,33^ In the other six studies, pharmacists were integrated as part of a multi-disciplinary team.^26–31^

The role of the pharmacist varied across studies, regardless of whether the study was pharmacist-led or part of a multi-disciplinary team. However, the primary focus consistently involved providing guidance on antibiotic dose optimization and adherence to therapeutic drug monitoring protocols.^24,25,27,29,31,32^ Wong *et al.*^28^ focused on the delivery of educational sessions for other healthcare professionals and the implementation of pharmacist-led drug management protocols. Epperson *et al.*^33^ reported pharmacist-led OPAT consultations prior to patient discharge, whereas other studies employed multi-disciplinary team consultations.^26,27^ A summary of the main characteristics of the included studies is presented in Table 1, while full study characteristics are provided in Table S1.

**Table 1.** Summary of included studies and reported outcomes

	Study characteristics			Clinical effectiveness		Complications			Cost-effectiveness
Year—Author—Country	Study design	Pharmacist role	Sample size	Laboratory monitoring adherence	Antimicrobial prescribing errors	Re-admissions	ED visits	Unfavourable outcomes	
2013—Keller *et al*—USA^26^	Pre- and post-implementation	Part of multi-disciplinary team	488	Yes	Yes	Yes	Yes	Yes	No
2015—Shah *et al*—USA^25^	Retrospective case–control	Pharmacist-led (second stage only)	106 (7 in second stage only)	Yes	No	No	No	No	No
2018—Hersh *et al*—USA^27^	Pre- and post-implementation	Part of multi-disciplinary team	776	No	No	No	No	Yes	No
2020—Howe *et al*—USA^32^	Retrospective observational cohort study	Pharmacist-led	117	No	No	Yes	No	No	No
2021—Wong *et al*—USA^28^	Retrospective pre- to post-cohort study	Part of multi-disciplinary team	323	No	No	Yes	No	Yes	No
2022—Thomnoi *et al*—Thailand^24^	Pre- and post-implementation	Pharmacist-led	100	Yes	Yes	No	No	Yes	No
2022—Hemenway *et al*—USA^29^	Retrospective case–control	Part of multi-disciplinary team	388	No	No	Yes	Yes	No	No
2023—Bellmeyer *et al*—USA	Pre- and post-implementation	Part of multi-disciplinary team	265	No	No	Yes	Yes	Yes	No
2023—Epperson *et al*—USA^33^	Retrospective cohort study	Pharmacist-led	399	No	No	Yes	Yes	No	Yes
2023—Missiaen *et al*—Belgium^31^	Retrospective cohort study	Part of multi-disciplinary team	85	No	No	Yes	No	Yes	No

### Methodological quality

The NOS scoring for each study can be found in Table S2. Three studies were rated as having a very high risk of bias^25,27,33^ and the other seven were rated as having a high risk of bias. Some studies had poor controls, for example, selecting their non-exposed cohort through identification of positive blood cultures only, while the intervention group was patients referred for an OPAT consultation.^26^ All studies were rated poor for adequacy of follow-up of their patients. In addition, most studies failed to adequately control for variables such as age, gender and co-morbidities.

## Study outcomes

### Clinical effectiveness

#### Therapeutic drug monitoring

Three studies specifically examined adherence to therapeutic drug monitoring and the adjustment of antibiotic doses.^24,25^ Two studies reported 100% adherence in their post-intervention groups,^24,25^ a considerable improvement from a baseline adherence rate of 60% and 35.9%, respectively. Keller and co-workers^26^ reported an improvement to 94.3% post-intervention from a 37.4% baseline. Meta-analysis was not possible due to the 100% outcome by two studies (Table S3).

It is important to note that the intervention group (Phase 2) in Shah and colleagues’s^25^ consisted of only seven patients, which limited the generalizability of the findings. In contrast, Thomnoi *et al.*'s^24^ study included 50 patients in each cohort and Keller *et al*.^26^ included 215 patients pre-intervention and 147 in the post-intervention, providing larger and more robust samples for comparison.

Hemenway and co-workers’^29^ study aimed to address three common failure points during transition of care to OPAT services, one of which was laboratory testing and review of results. This involved both the nurse and pharmacist working together to proactively track when tests were required and review the results weekly as part of their interventions. However, no quantitative results for this outcome were given.

#### Prescribing errors

The definition of prescribing errors varied between studies, ranging from transcription discrepancies between discharge summaries and infectious diseases recommendations to inappropriate antimicrobial dosing. Keller *et al.*^26^ defined errors as differences between the antimicrobial agent written on the discharge summary and the infectious diseases consult service management plan. Thomnoi *et al.*^24^ defined prescribing errors as inappropriate antimicrobial dosing, for which they recorded the number of recommendations made by the clinical pharmacist and the percentage accepted by physicians.

Prescribing errors reduced from 18.1% pre-intervention to 1.36% post-intervention (OR 0.06, 95% CI 0.02–0.26) in the study by Keller *et al.,*^26^ which was associated with improvements in readmissions, ED visits, and complications in the same study. Thomnoi *et al.*^24^ also reported a reduction in prescribing errors from 22% pre-intervention to 0% post-intervention.

Owing to differences in definitions and outcome reporting, including one study reporting a 0% post-intervention outcome, meta-analysis was not feasible (Table S3).

### Complications

#### Re-admissions

**Figure 2.**
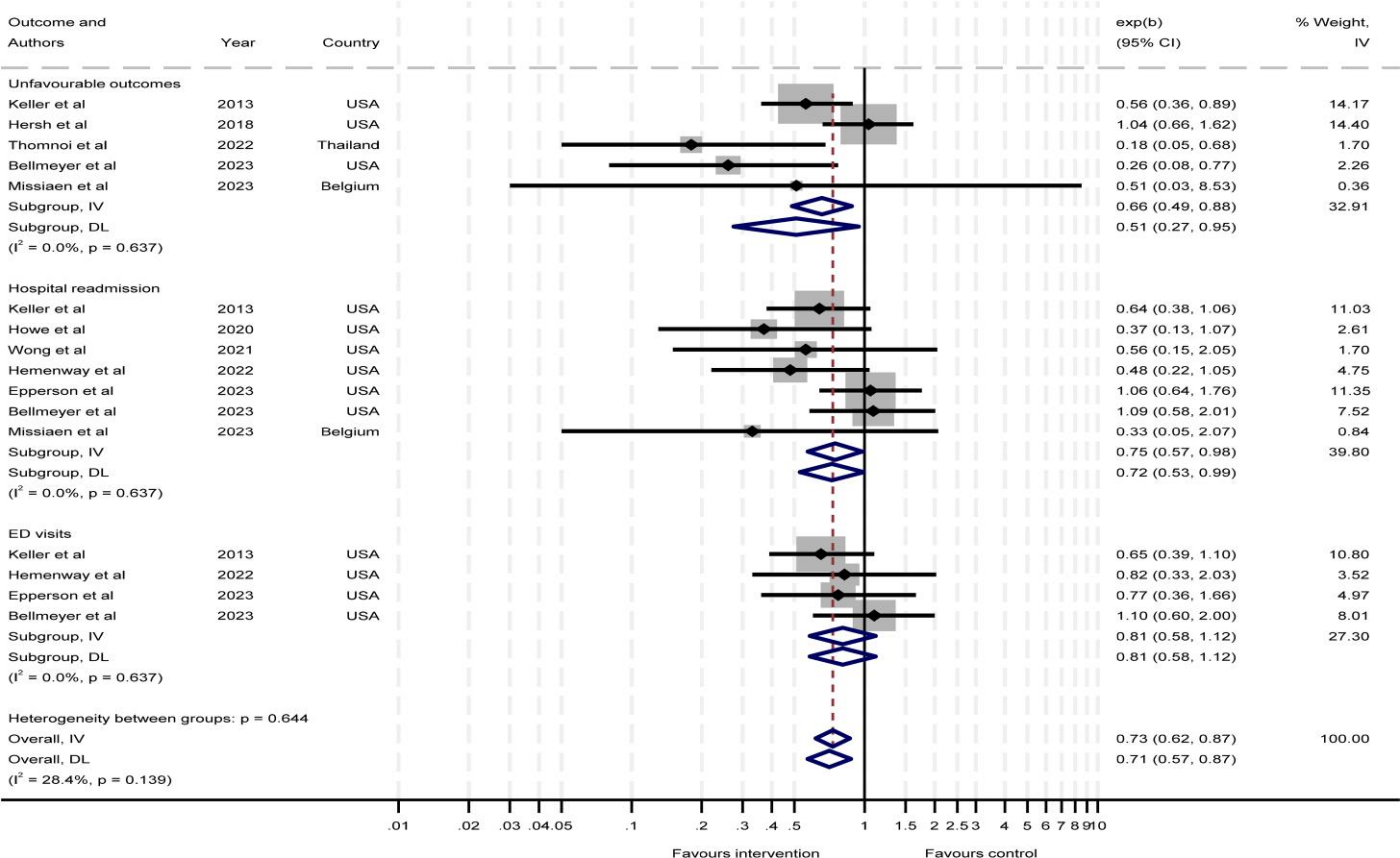
Meta-analysis of studies included in systematic review.

Seven studies assessed hospital readmission during OPAT, with a mean follow-up of approximately 30 days.^26,28–34^ Although no individual study demonstrated a statistically significant reduction in readmissions, meta-analysis showed a statistically significant reduction associated with the interventions overall (OR 0.75, 95% CI 0.57–0.98) (Figure 2). Two of the included studies evaluated pharmacist-led OPAT services, while others were involved in multi-disciplinary teams.

There was variation in definitions of readmission and follow-up duration across studies. Some studies reported all-cause or infection-related readmissions without a defined follow-up period,^28–31,33^ while others specified a 30-day follow-up but did not consistently define readmission as OPAT-related or infection-related.^26,32,34^

#### Visits to ED during OPAT

Four studies reported ED attendance as an outcome.^26,29,30,33^ Three studies reported ED visits related to OPAT or infection-related causes,^29,30,32^ while Keller *et al.*^26^ did not specify the indication for ED attendance.

Pooled analysis showed a 19% reduction in ED attendance associated with the interventions (OR 0.81, 95% CI 0.58–1.12) (Figure 2); however, this did not reach statistical significance. All included studies involved pharmacist participation as part of a multi-disciplinary team.

#### Unfavourable outcomes

Five studies included unfavourable outcomes.^24,26,27,30,31^ The odds of unfavourable outcomes were 34% lower with intervention (OR 0.66, 95% CI 0.49–0.88) (Figure 2), one evaluated a pharmacist-led intervention, while pharmacists formed part of the multi-disciplinary team in five studies.

The studies reported different aspects of care under unfavourable outcomes, making comparison and grouping of results challenging. Thomnoi and co-workers^24^ focused on worsening infection or hospital mortality whilst Missiaen and colleagues^31^ reported mortality as a separate outcome. Three studies addressed vascular access device (VAD) issues, with Billmeyer *et al.*^30^ grading adverse events by severity but without defining the scale. Keller *et al.*^26^ included adverse antimicrobial events, catheter complications, and infection relapse but did not clarify assessment methods or severity grading. They also reported *Clostridium difficile* infection rates as a distinct outcome. Hersh *et al.*^27^ defined OPAT complications as unplanned ED visits or hospitalizations due to line-related problems.

### Cost-effectiveness

One study reported cost savings associated with re-admissions. Epperson and colleagues^33^ performed a post hoc stratified analysis for patients discharged specifically with vancomycin. Patients discharged with an OPAT monitoring programme showed significantly lower readmission rates (19.4% versus 39.1%; *P* = 0.004), which were then quantified into cost of each re-admission ($18 872). Therefore, a cost-saving of $339 690 was predicted as a potential saving if all patients in the intervention and control cohorts were included. No other study included the cost savings reported due to the interventions made.

## Discussion

Our systematic review identified ten studies from 2013 to 2023, which involve pharmacists in OPAT services, either as a pharmacist-led service or part of a multi-disciplinary team with quantifiable outcomes. The findings of the individual studies included indicate that the involvement of pharmacists was associated with a significant improvement in process outcomes such as adherence to drug monitoring protocols and the optimization of drug dosing. The meta-analysis demonstrated a significant reduction in unfavourable outcomes and hospital readmissions across included interventions, which comprised both pharmacist-led services and multi-disciplinary OPAT teams involving pharmacists.

OPAT services have developed over the past five decades and now represent an important component of AMS infrastructure, supported by multi-disciplinary infection teams globally. A systematic review by Dighiriri *et al.* described a wide range of AMS activities undertaken by pharmacists across diverse geographical and healthcare settings, including audit and feedback, optimization of therapy and education of healthcare professionals and patients.^34^ Within OPAT services, the Society of Infectious Diseases Pharmacists (SIDP) recognizes antimicrobial pharmacists as key members of AMS programmes across different settings however acknowledges that many institutions have not implemented this despite recommendations by the Infectious Diseases Society of America (IDSA).^10,35^

It is also recognized that OPAT services may be practiced differently depending on factors such as geography, availability of specialist services and the maturity of AMS and OPAT services within different countries.^36^ A scoping review explored the role of community pharmacies and AMS intervention, which identified a growing but limited evidence base within this area, despite most studies being conducted in high-income countries but excluded outpatient settings.^37^

BSAC OPAT good practice recommendations, while primarily developed for UK practice, were used as a structured framework for interpretation due to their detailed recommendations regarding OPAT service structure and AMS principles. These recommendations are broadly consistent with international guidance, such as the IDSA, given the majority of included studies were conducted in the United States.

### Clinical effectiveness

Only three studies explored clinical effectiveness outcomes such as adherence to antimicrobial guidelines and therapeutic drug monitoring in OPAT settings. This is of note, given that many of the antimicrobials used within the studies required dose adjustments on patient-specific factors such as renal function or therapeutic levels, suggesting that monitoring and dosing practices would be expected to feature more prominently in outcome reporting. For example, gentamicin and vancomycin, included in six of the studies in this review, are nephrotoxic meaning, they require regular therapeutic drug monitoring and dose adjustment.^38–40^ However, it was not consistently reported whether monitoring and dose optimization occurred prior to discharge or during OPAT follow-up nor which healthcare professional was responsible for undertaking these activities within the included studies. This is despite monitoring and dose adjustment being established components of pharmacist roles in non-OPAT settings.^41^

Within this systematic review, pharmacist interventions were shown to reduce antimicrobial prescribing errors, reporting absolute risk reductions in errors of 17%–22%.^24,26^ A major limitation of the included studies was the definition of prescribing errors, ranging from transcription errors to guideline adherence, which made comparison difficult. However, this pharmacist role is consistent with evidence from other hospital-based settings, including ED.^42,43^ In neonates, pharmacist interventions reduced antibiotic use rates by 23% in a systematic review and meta-analysis by Lee and An.^44^

Additionally, in OPAT settings, pharmacists have been shown to contribute to antimicrobial selection, treatment duration decisions, and therapeutic drug monitoring.^45^ Hino *et al.* reported modelled benefits, including earlier intravenous-to-oral switch and optimization of antimicrobial regimens suitable for OPAT delivery.

### Complications

In this systematic review and meta-analysis, observed effects on patient outcomes, hospital re-admissions, ED visits and complications varied. Only three of the studies showed significant effects favouring interventions for re-admissions and unfavourable outcomes.^24,26,29^ The remaining studies reported no significance regardless of whether it was a pharmacist-led or MDT-led intervention programme, but this may have been due to the small sample sizes and lack of statistical significance to detect a difference.

This aligns with previous systematic reviews, which highlight the importance of antimicrobial pharmacist interventions on reducing antimicrobial use, cost and clinical processes, but their effect on health outcomes remains difficult to directly measure despite the evolution in the role.^46^

### Cost effectiveness

In this systematic review, only one study measured cost-effectiveness of interventions made. However, while OPAT as a service has been previously demonstrated to be a cost-effective use of resources,^47^ health economic analysis of AMS intervention strategies were shown to be a critical gap worthy of further research.^48^

### Limitations

One of the most notable limitations of this review is the high risk of bias of the included studies. However, no studies were excluded from the analysis on the basis of quality, due to the absence of high-quality studies and relatively small overall number of studies identified. This increased the risk of bias in the findings of this review overall. Ideally, a randomized controlled trial design would eliminate much of the selection bias observed to date. Researchers should consider reporting more detail regarding the antimicrobial agents used, such as the route and duration of therapy following the TiDier checklist or similar.^49^

In addition, there was significant variability in outcome definitions, follow-up durations, and infection complexity across studies, which limited comparability and made meaningful interpretation of results difficult. The grouping of simple and complex infections, like UTIs and endocarditis, further reduced the reliability of outcome interpretations. The BSAC National Outcomes Registry is a voluntary database which collected outcome data with standardized definitions in the UK, researchers should consider a similar data collection mechanism.^50^

The majority of included studies were conducted in the United States, with one study from Thailand and one from Belgium. This geographic distribution may limit the generalizability of the findings, as the structure, maturity of AMS programmes, and organization of OPAT services vary significantly between healthcare systems.

Further limitations include the use of a single researcher for abstract screening and data extraction, which may increase the risk of selection and extraction bias. Additionally, qualitative studies and conference abstracts were excluded from the search strategy, as they would not have been amenable to quantitative analysis or formal risk-of-bias assessment, respectively. Future updates of this systematic review should consider incorporating these sources using appropriate methodological approaches.

### Recommendations

Based on the findings of this systematic review, several areas require further investigation to better assess current practice and support the development of OPAT services:

- Future studies should include larger patient cohorts to improve statistical power and generate more clinically meaningful and generalizable findings.
- There is a need for further research evaluating pharmacist interventions specifically within OPAT settings, as evidence remains limited despite established effectiveness in other clinical environments and alignment with BSAC and IDSA recommendations.
- The development and adoption of a core outcome set for OPAT research is recommended to standardize reporting. Key outcomes should include hospital readmissions, emergency department visits, and treatment-related complications to improve comparability across studies.
- Future studies should incorporate longer follow-up periods to better capture outcomes in patients with complex infections managed in OPAT settings.
- Clear reporting of patient follow-up is required, including documentation of loss to follow-up and, where possible, the reasons for attrition, to improve transparency and reduce risk of bias.

## Conclusions

The findings of this systematic review suggest that pharmacist involvement in OPAT settings may improve adherence to antibiotic laboratory monitoring guidelines and reduce antimicrobial prescribing errors. In addition, the meta-analysis demonstrated a reduction in unfavourable outcomes and re-admissions which had not been detected in smaller studies.

## Supplementary Material

dkag237_Supplementary_Data

## Supplementary data

Appendix S1 and Tables S1 to S3 are available as Supplementary data at *JAC* Online.
