# Supplementary material for: Pharmacists in OPAT: a systematic review and meta-analysis
Source: J Antimicrob Chemother. 2026 Jul 15;81(8):dkag237. doi: 10.1093/jac/dkag237 (PMC13370828; doi:10.1093/jac/dkag237)
Supplement: dkag237_Supplementary_Data [file dkag237_supplementary_data.docx]

**Appendix 1: Search Strategy for Web of Science and Embase**

Web of Science:

(ALL=("antimicrobial stewardship pharmacist" OR "AMS pharmacist" OR "ID pharmacist" OR "infectious disease pharmacist" OR "ID pharmacy" OR "clinical pharmacy" OR "pharmacist" OR "hospital pharmacist" OR "pharmacy") AND ALL=("pharmacist-led" OR "pharmacist intervention" OR "pharmacist managed") AND ALL=("outpatient parenteral antimicrobial therapy" OR "OPAT" OR "outpatient parenteral antibiotic therapy" OR "HIVAT" OR "hospital in the home" OR "hospital at home") AND ALL=("antibiotic" OR "antiinfective" OR "antimicrobial" OR "anti-infective"))

Embase search:

(OPAT OR 'outpatient parenteral antibiotic therapy'/exp OR 'outpatient parenteral antimicrobial therapy'/exp OR 'home intravenous therapy'/exp OR 'outpatient parenteral antibiotic therapy' OR 'outpatient parenteral antimicrobial therapy' OR HIVAT OR OHPAT) AND ('pharmacist intervention'/exp OR pharmacist-led OR 'pharmacist managed' OR ('clinical pharmacy'/exp OR 'clinical pharmacy' OR 'pharmacy, clinical') OR 'antimicrobial pharmacist' OR 'ID Pharmacist' OR ('hospital pharmacy'/exp OR 'clinical pharmacy service' OR 'hospital pharmaceutic service' OR 'hospital pharmaceutical service' OR 'hospital pharmaceutical services' OR 'hospital pharmacies' OR 'hospital pharmacy' OR 'hospital pharmacy service' OR 'pharmacy service, hospital') OR 'hospital pharmacy' OR pharmacist) AND (antimicrobials/exp OR ('antiinfective agent'/exp OR 'anti bacterial agent' OR 'anti bacterial agents' OR 'anti infective agents' OR 'anti infectives, otic' OR 'anti-bacterial agents' OR 'anti-infective agents' OR 'anti-infectives, otic' OR 'antibacterial' OR 'antibacterial agent' OR 'antibacterial drug' OR 'antibacterial soap' OR 'antibacterial spectrum' OR 'antiinfective agent' OR 'antiinfectives, otic' OR 'antimicrobial' OR 'antimicrobial agent' OR 'antimicrobial compound' OR 'antimicrobial drug' OR 'antimicrobial factor' OR 'antiseptic' OR 'antiseptic agent' OR 'antiseptic cream' OR 'antiseptic foam' OR 'antiseptic soap' OR 'chemotherapeutic agent' OR 'chemotherapeutic drug' OR 'chemotherapeutica' OR 'microbiological agent') OR antiinfective OR ('antibiotic agent'/exp OR 'antibiotic' OR 'antibiotic agent' OR 'antibiotic combination' OR 'antibiotic drug' OR 'antibiotic ointment' OR 'antibiotic residue' OR 'antibiotic spectrum' OR 'antibiotics' OR 'antibiotics and their derivatives' OR 'antibiotics, combined' OR 'antibiotics, folate antagonists' OR 'antibiotics, miscellaneous' OR 'antibiotics, nitrofuran' OR 'antibiotics, oxalodinones' OR 'combined antibiotic') OR antimicrobials)

**Table S1: Study characteristics included studies**

| *Study Characteristics* | | | | | | | | | |
| --- | --- | --- | --- | --- | --- | --- | --- | --- | --- |
| Year - Author - Country | Title | Study design | Type of OPAT setting | Patient characteristics and sample size | Infections treated | Antibiotics used | Pharmacist intervention | Primary outcomes | Secondary outcomes |
| 2013 – Keller et al - USA | The Impact of an Infectious Diseases Transition Service on the Care of Outpatients on Parenteral Antimicrobial Therapy | Pre and post implementation | Home or skilled nursing facility | Mean age 56.4 ± 16.0 years. 40% female (n=488) | Details of pre- and post-implementation were grouped: Bacteraemia (35.9%), osteomyelitis/septic arthritis (22.7%), endocarditis or vascular (16.8%), neurologic infection (10.2%), abdominal abscess (7.38%), skin or soft tissue abscess or cellulitis (3.89%) | Vancomycin (37.8%), penicillin (30.3%), cephalosporin (25.4%), carbapenem (5.12%), aminoglycoside (4.71%), daptomycin (3.67%), antifungal agent 3.48%), other agents (1.43%) | Consultation by ID team and co-ordinated follow up by ID physician or pharmacist | Readmissions, ED visits, and mortality within 60 days of discharge | ALL: readmissions and/or ED visits within 7 days of discharge and within 30 days of discharge. Deaths within 60 days of discharge. Intervention only: adverse antimicrobial events, catheter complications, infection relapse, and Clostridium difficile infections, process of care measures and non-readmission clinical outcomes |
| 2015 - Shah et al - USA | Monitoring of Outpatient Parenteral Antimicrobial Therapy and Implementation of Clinical Pharmacy Services at a Community Hospital Infusion Unit | Retrospective case control | Infusion centre | Mean age 63.1 years ± 16.6 years. 63% female (n=106) | Non-ID physician: Urinary tract infections (46.7%), skin and soft tissue infection (20%), bacteraemia (13.3%), healthcare associated pneumonia (8.9%), community associated pneumonia (6.7%), osteomyelitis (2.2%), chronic sinusitis (2.2).   ID physician: Skin and soft tissue infection (32.8%), urinary tract infections (19.7%), bacteraemia (13.1%), intra-abdominal infections (9.8%), osteomyelitis/ prosthetic joint infections (8.2%), endocarditis (3.3%), healthcare associated pneumonia (3.3%), chronic sinusitis (3.3%), meningitis (1.6%), syphilis (1.6%). | Non-ID physician: Ceftriaxone (25.6%), cefepime (18.6%), gentamicin (16.3%), ertapenem (11.6%), vancomycin (11.6%), tobramycin (4.7%), daptomycin (4.7%), linezoliden3%), azithromycin (2.3%), meropenem (2.3%).  ID physician: Vancomycin (19.7%), daptomycin (18.2%), ertapenem (16.7%), ceftriaxone (12.1%), cefepime (10.6%), gentamicin (10.6%), tobramycin (4.5%), benzathine penicillin G (1.5%), ceftazidime (1.5%), tigecycline (1.5%), linezolid (1.5%), azithromycin (1.5%) | Pharmacist monitoring of OPAT prescribed by non-ID physicians | Adherence to guidelines on monitoring. Attainment of goal vancomycin and aminoglycoside serum concentrations | None |
| 2018 – Hersh et al   - USA | Impact of Antimicrobial Stewardship for Pediatric Outpatient Parenteral Antibiotic Therapy | Pre and post implementation | Home | Paediatrics – age and gender not stated (n=776) | Details of pre- and post-implementation were grouped: Fever/neutropenia, bloodstream infections, osteoarticular infection, surgical site infection, respiratory infections (exact numbers not stated) | No detail of antibiotics used provided | Pharmacist as part of stewardship team reviews/makes recommendations prior to discharge | Number of patients discharged with OPAT and reviewed by stewardship team/had undergone ID consultation | Number of stewardship recommendations, OPAT indications, OPAT complications (unplanned emergency department visit or hospitalization for line clot, dislodgement, or infection) compared with those discharged with oral antimicrobials and re-admitted within 30 days |
| 2020 – Howe et al - USA | Comparison of Patient Outcomes in a Pharmacist-Led Parenteral Antimicrobial Therapy Program | Retrospective observational cohort study | Community hospital | Median age 61 years, IQR 21 (OPAT group) and 64 years IQR 26.3 (no OPAT consult). Gender not stated (n=117) | No OPAT consult: Empyema (0.09%), osteomyelitis (0.09%), bacteraemia (59%), intra-abdominal infection (14%), other (0.09%). OPAT consult: Empyema (7.4%), osteomyelitis (11.6%), bacteraemia (37.9%), intra-abdominal infection (10.5%), skin and soft tissue infection (27.3%), other (5.3%) | Anti-pseudomonal coverage (14.74% in OPAT group vs 38.95% in no OPAT consult). Ceftriaxone (9.47% in OPAT group vs 45.54% in no OPAT consult). Vancomycin (41.05% in OPAT group vs 86.36% in no OPAT consult). Gentamicin (6.32% in OPAT group vs 22.73% in no OPAT consult) | Pharmacist responsibilities to evaluate and create a plan with recommendations related to antimicrobial selection (including drug, dose, route, frequency, and duration) as well as monitoring parameters. Provide patient education and assistance to case managers involved with disposition planning. On discharge, the pharmacist continued weekly monitoring throughout the duration of therapy of all patients who received such consultative services during their inpatient stay. | proportion of patients in each group readmitted within 30 days of discharge and OPAT initiation, which was stratified by the reason for readmission (ID process, adverse drug event, or unrelated reason). | Collection of data including age, sex, weight and length of stay prior to discharge. Type of infection, antimicrobial selection (including agents with antipseudomonal activity or requiring therapeutic drug monitoring), duration of treatment, and disposition at hospital discharge were also collected from the electronic medical record. Change in disposition from admission data was also collected |
| 2021 – Wong et al - USA | Retrospective assessment of antimicrobial stewardship initiative in outpatient use of ertapenem for uncomplicated extended spectrum beta lactamase Enterobacteriaceae urinary tract infections | Retrospective pre to post cohort study | Infusion centre, urgent care department, emergency department or home | Mean age 67 years ± 17 years. Females > 18 years (n=323) | Uncomplicated extended spectrum beta lactamase Enterobacteriaceae urinary tract infections | Amikacin (38% pre-intervention vs 45% post-intervention). Gentamicin (44% pre-intervention vs 43% post-intervention). Culture sensitivity unknown (18% pre-intervention vs 12% post-intervention) | Educational sessions to targeted towards medical specialties including hospitalist, emergency medicine/urgent care physicians, intensivists, and pharmacists. Pharmacist-led drug use management protocol | clinical resolution defined as cure, persistence, relapse or recurrence | monthly ertapenem use (days of therapy/1000 adjusted bed days) |
| 2022 - Thomnoi et al  - Thailand | Impact of Pharmacist-Led Implementation of a Community Hospital-Based Outpatient Parenteral Antimicrobial Therapy on Clinical Outcomes in Thailand | Pre and post implementation | Community hospital infusion centre | Median age 63 years (pre-implementation), 66.5 years (post-implementation). 38% female (pre-implementation), 48% female (post-implementation) (n=100) | Pre-implementation: Lower respiratory infections (54%), bloodstream (26%), urinary tract (14%), intra-abdominal (10%), skin and soft tissue (6%), osteoarticular (6%), central nervous system (2%), cardiovascular (2%).   Post-implementation: lower respiratory infections (30%), bloodstream (38%), urinary tract (26%), intra-abdominal (10%), skin and soft tissue (22%), osteoarticular (2%), central nervous system (2%), cardiovascular (0%) | Not stated | Adherence to clinical practice guidelines and medication management | Percentage of patients with lab monitoring complying with clinical practice guidelines and doses adjusted accordingly | Number of medical related problems verified by a clinical pharmacist. Percentage of pharmacist recommendations considered accepted by physicians |
| 2022 - Hemenway et al  - USA | Reflections on implementation of a failure-point–focused outpatient parenteral antimicrobial therapy management program | Retrospective case control | OPAT clinic | Mean age and gender similar between both groups but numbers not stated (n=388) | Not stated | Not stated | Review of lab results and monitoring. Follow up at the end of planned course to ensure antibiotics stopped and PICC line removed | (1) patients who leave the facility on inappropriate IV antimicrobials, without weekly laboratory test orders, or no follow up appointment, (2) delayed assessment of held virtually weekly laboratory results (3) antimicrobials not stopped as planned and/or peripherally inserted central catheter (PICC) not removed at the end of therapy | None |
| 2023 - Bellmeyer et al - USA | Predictors of adverse safety events and unscheduled care among an outpatient parenteral antimicrobial therapy (OPAT) patient cohort | Pre and post implementation | Home with Fairview home infusion service | Median age 51.2 years (IQR 37.9). 48% female (n=265) | Details of pre- and post-implementation were grouped: Bloodstream infections, including candidemia (33.2%), bone and joint (18.1%), skin and soft tissue (16.2%), intra-abdominal (14.3%), genitourinary (12.8%), bacterial pneumonia (6.4), other (5.7%), endocarditis (4.9%), bacterial central nervous system (3.4%), viral (3%), fungal excluding candidemia (1.5%) | Cephalosporin (61.1%), Carbapenem (21.5%), Lipo/glycopeptide (16.2%) Penicillin (6.8%), Beta-lactam/beta-lactamase inhibitor (6.4%),  Pyrophosphate analogue (2.3%),  Echinocandin (2.3%), Aminoglycoside (1.1%), Azole (0.8%), Lincosamide (0.8%), Nucleoside analogue (0.8%),  Monobactam (0.4%), Nitroimidazole (0.4%) | Medicine reconciliation, antibiotic dose optimisation | predictors of vascular access device (VAD) complications, adverse drug events and OPAT-related emergency department visits and rehospitalisation | None |
| 2023 - Epperson et al  - USA | Impact of a Pharmacist-Managed Outpatient Parenteral Antimicrobial Therapy (OPAT) Service on Cost Savings and Clinical Outcomes at an Academic Medical Center | Retrospective cohort study | Home | Median age 54 (IQR 21). 47% female (n=399) | Control: Bone/joint (23%), bone/joint + hardware (6%), central nervous system (3%), bacteraemia (37%), skin/skin structure (25%), urinary tract (15%), pneumonia (8%), intra-abdominal (8%).  OPAT: Bone/joint (29%), bone/joint + hardware (11%), central nervous system (7%), bacteraemia (31%), skin/skin structure (31%), urinary tract (12%), pneumonia (3%), intra-abdominal (6%) | Control: Vancomycin (15%), ceftriaxone (21%), piperacillin-tazobactam (18%), cefazolin (13%), linezolid (15%), cefepime (6%).  OPAT: Vancomycin (38%), ceftriaxone (14%), piperacillin-tazobactam (13%), cefazolin (8%), linezolid (7.4%), cefepime (7%) | OPAT pharmacist-led consultation prior to discharge | All cause re-admission within 30 days of discharge | infection-related readmission within 30 days of discharge, all-cause and infection-related readmission within 3 days of discharge, hospital length of stay during index admission and readmission, and infection-related emergency department (ED) visits within 60 days of discharge. |
| 2023 - Missiaen - Belgium | Retrospective evaluation of an intervention bundle on OPAT implementation in a large non-university hospital | Retrospective cohort study | Home | Pre-intervention 59 (±17.8) 24% female, post intervention: 63 (±17.1) 37% female (n=85) | Pre-intervention: UTI 36%, prostatitis 9%, cystitis 0%, pyelonephritis 0%, recurrent UTI 6%, other urinary tract infections 21%, prosthetic joint infection 3%, Spondylodiscitis 3%, osteomyelitis 3%, Bacteremia 21%, endocarditis 3%, Abdominal infections 3%, Skin/soft-tissue infection 6%, CNS infection 3%, COPD exacerbation 3%, pneumonia 3%, other 3%.   Post-intervention: UTI 25%, prostatitis 4%, cystitis 3%, pyelonephritis 5%, recurrent UTI 2%, other urinary tract infections 2%, prosthetic joint infection 14%, Spondylodiscitis 11%, osteomyelitis 10%, Bacteremia 8%, endocarditis 10%, abdominal infections 3%, Skin/soft-tissue infection 5%, CNS infection 5%, COPD exacerbation 2%, pneumonia 3%, other 3% | Pre-intervention: Ceftriaxone 24%, Temocillin 33%, Teicoplanin 6%, Meropenem 9%, ceftazidime 15%, piperacillin-tazobactam 3%, cefepime 3%, aciclovir 3%, cefazolin 3%, amoxicillin 0%, aztreonam 0%, flucloxacillin 0%, penicillin 0%, tigecycline 0%  Post-intervention: Ceftriaxone 27%, Temocillin 19%, Teicoplanin 25%, Meropenem 13%, ceftazidime 2%, piperacillin-tazobactam 5%, cefepime 2%, aciclovir 0%, cefazolin 0%, amoxicillin 2%, aztreonam 2%, flucloxacillin 2%, penicillin 2%, tigecycline 2% | revision of the internal OPAT guidelines and procedures including standardized procedure of preparing and delivering antibiotics and administration sets, as well as improving the method of intravenous administration from continuous infusion to bolus administration as part of MDT, attending weekly multidisciplinary staff meeting for orthopedic and prosthetic joint infections. Reorganization of the OPAT delivery process in the pharmacy, addressing the practical issues of prescription, and distribution of both parenteral antimicrobials and standardized administration sets; and implementation of a revised consensus protocol with state-of-the-art guidelines for optimal and standardized diagnosis and treatment of PJI | the amount of OPAT trajectories initiated before and after the intervention,  6-month mortality rate following the OPAT-treatment, mortality rate during OPAT treatment, readmission rate during treatment and its relationship with OPAT, and recurrence of infections within 6 months after discontinuation of OPAT as a proxy for cure. | None |

**Table S2: Risk of Bias assessed using Newcastle-Ottowa score**

|  | Newcastle-Ottowa Score | | | | | | | | | Total | Comments | |  |  |
| --- | --- | --- | --- | --- | --- | --- | --- | --- | --- | --- | --- | --- | --- | --- |
| Author, Year | 1 | 2 | 3 | 4 | 5 | | 6 | 7 | 8 |  |  |  |  |  |
| Keller, 2013 | 0 | 0 | 1 | 1 | 1 | | 1 | 1 | 0 | 5 | Both intervention and control group were not a true representation of the average patient discharged with OPAT. Control population only identified through positive blood cultures, only patients with a requested OPAT consultation included. No mention of adequacy of follow up of cohorts | |  |  |
| Shah, 2015 | 0 | 0 | 0 | 1 | 0 | | 0 | 0 | 0 | 1 | Both intervention and control group were not true representative of the average patient discharged with OPAT, no mention of ascertainment of exposure, no adjustment made for controls. No description of follow up duration or adequacy of follow up of cohorts. | |  |  |
| Hersh, 2017 | 1 | 1 | 0 | 1 | 0 | | 0 | 0 | 0 | 3 | No description of ascertainment of exposure, no adjustments made for controls, assessment of exposure was self-reported, follow up duration not mentioned, adequacy of follow up of cohorts not mentioned | |  |  |
| Howe, 2020 | 1 | 1 | 0 | 1 | 1 | | 0 | 1 | 0 | 5 | No description of ascertainment of exposure, no description of assessment of outcome, adequacy of follow up of cohorts not mentioned | |  |  |
| Wong, 2021 | 0 | 1 | 1 | 1 | 1 | | 1 | 0 | 0 | 5 | Both intervention and control group were not a true representation of the average patient discharged with OPAT, as only females with uncomplicated ESBL UTIs included. No description of assessment of outcome and adequacy of follow up of cohorts not mentioned | |  |  |
| Hemenway, 2022 | 1 | 1 | 1 | 1 | 0 | | 0 | 0 | 0 | 4 | No mention of controls for study variables, no description of assessment of outcome, no mention of duration of follow up or adequacy of follow up of cohorts. | |  |  |
| Thomnoi, 2022 | 1 | 1 | 1 | 0 | 2 | | 1 | 0 | 0 | 6 | Patients who died within 24 hours of admission were excluded, no mention of duration of follow up or adequacy of follow up of cohorts | |  |  |
| Billmeyer, 2023 | 1 | 1 | 1 | 0 | 1 | | 1 | 1 | 0 | 6 | Outcomes of interest e.g. VAD complications or adverse effects could have been present at start. No mention of adequacy of follow up of cohorts | |  |  |
| Epperson, 2023 | 1 | 0 | 0 | 1 | 0 | | 0 | 0 | 0 | 2 | Intervention and control groups were picked from different cohorts, ascertainment of exposure was only from PICC log enough which was not sufficient. Although similar rates of characteristics between control and intervention group, no adjustment made for study controls, outcome data was obtained at one institute only. 30 day re-admission was deemed to be insufficient if patients are given prolonged courses of antibiotics via OPAT, no mention of adequacy of follow up of cohorts | |  |  |
| Missiaen, 2023 | 1 | 1 | 0 | 1 | 0 | | 0 | 1 | 0 | 4 | No controls for age or additional factors. No description of ascertainment of exposure or how outcome of interest were obtained. No mention of adequacy of follow up. | |  |  |
| **Selection**   1. **Representation of the average patient discharged with OPAT** 2. **Selection of the non-exposed cohort** 3. **Ascertainment of exposure** 4. **Demonstration that the outcome of interest was not present at the start of the study** | | | | | | | **Comparability**   1. **Comparability of cohorts on the basis of the design or analysis:**   **1 point – study controls for age**  **2 points = study controls for additional factors (gender, co-morbidities)** | | | | | | **Outcome**   1. **Assessment of outcome** 2. **Was the follow up long enough for the outcome to occur** 3. **Adequacy of follow up of cohorts** | |

**Table S3: Study outcomes of included studies**

| Study Characteristics | | | | Clinical effectiveness | | Complications | | | Cost effectiveness |
| --- | --- | --- | --- | --- | --- | --- | --- | --- | --- |
| Year - Author - Country | Title | Type of pharmacist involvement | Sample size | Laboratory monitoring adherence | Antimicrobial prescribing errors | Re-admissions | ED visits | Unfavourable outcomes |  |
| 2013 – Keller et al - USA | The Impact of an Infectious Diseases Transition Service on the Care of Outpatients on Parenteral Antimicrobial Therapy | Part of multi-disciplinary team | 488 | OR 0.06 (95% CI 0.03-0.11)^[[1]](#footnote-1)^ | OR 0.06 (95% CI 0.02-0.26)^[[2]](#footnote-2)^ | 0.64 (OR, 95% CI 0.38-1.06)^[[3]](#footnote-3)^ | 0.65 (OR, 95% CI 0.39-1.10)^[[4]](#footnote-4)^ | 0.56 (OR, 95% CI 0.36-0.89)^[[5]](#footnote-5)^ | No |
| 2015 - Shah et al - USA | Monitoring of Outpatient Parenteral Antimicrobial Therapy and Implementation of Clinical Pharmacy Services at a Community Hospital Infusion Unit | Pharmacist-led (2^nd^ stage only) | 106 (7 in 2^nd^ stage only) | 35.9% (pre-intervention) to 100% (post-intervention)^[[6]](#footnote-6)^ | No | No | No | No | No |
| 2018 – Hersh et al   - USA | Impact of Antimicrobial Stewardship for Pediatric Outpatient Parenteral Antibiotic Therapy | Part of multi-disciplinary team | 776 | No | No | No | No | 1.04 (OR, 95% CI 0.66-1.62)^[[7]](#footnote-7)^ | No |
| 2020 – Howe et al - USA | Comparison of Patient Outcomes in a Pharmacist-Led Parenteral Antimicrobial Therapy Program | Pharmacist-led | 117 | No | No | 0.37 (OR, 95 % CI 0.13-1.07)^[[8]](#footnote-8)^ | No | No | No |
| 2021 – Wong et al - USA | Retrospective assessment of antimicrobial stewardship initiative in outpatient use of ertapenem for uncomplicated extended spectrum beta lactamase Enterobacteriaceae urinary tract infections | Part of multi-disciplinary team | 323 | No | No | 0.56 (OR, 95% CI 0.15-2.05)^[[9]](#footnote-9)^ | No | Pre-intervention 3% vs post intervention 0%^[[10]](#footnote-10)^ | No |
| 2022 - Thomnoi et al - Thailand | Impact of Pharmacist-Led Implementation of a Community Hospital-Based Outpatient Parenteral Antimicrobial Therapy on Clinical Outcomes in Thailand | Pharmacist-led | 100 | 60% (pre-intervention) to 100% (post-intervention)^[[11]](#footnote-11)^ | 22% (pre-intervention) to 0% (post-intervention)^[[12]](#footnote-12)^ | No | No | 0.18 (OR, 95% CI 0.05-0.69)^[[13]](#footnote-13)^ | No |
| 2022 - Hemenway et al  - USA | Reflections on implementation of a failure-point–focused outpatient parenteral antimicrobial therapy management program | Part of multi-disciplinary team | 388 | No | No | 0.48 (OR, 95% CI 0.22-1.05)^[[14]](#footnote-14)^ | 0.82 (OR, 95% CI 0.33-2.03)^[[15]](#footnote-15)^ | No | No |
| 2023 - Bellmeyer et al - USA | Predictors of adverse safety events and unscheduled care among an outpatient parenteral antimicrobial therapy (OPAT) patient cohort | Part of multi-disciplinary team | 265 | No | No | 1.09 (OR, 95% CI 0.58–2.01)^[[16]](#footnote-16)^ | 1.1 (OR, 95% CI 0.60–2.00)^[[17]](#footnote-17)^ | 0.26 (OR, 95% CI 0.08–0.77)^[[18]](#footnote-18)^ | No |
| 2023 - Epperson et al  - USA | Impact of a Pharmacist-Managed Outpatient Parenteral Antimicrobial Therapy (OPAT) Service on Cost Savings and Clinical Outcomes at an Academic Medical Center | Pharmacist-led | 399 | No | No | 1.06 (OR, 95% CI 0.64-1.76)^[[19]](#footnote-19)^ | 0.77 (OR, 95% CI 0.36-1.66)^[[20]](#footnote-20)^ | No | $18,872^[[21]](#footnote-21)^ |
| 2023 - Missiaen - Belgium | Retrospective evaluation of an intervention bundle on OPAT implementation in a large non-university hospital | Part of multi-disciplinary team |  | No | No | 0.33 (OR, 95% CI 0.05-2.07)^[[22]](#footnote-22)^ | No | 0.51 (OR 95% CI 0.03-8.53)^[[23]](#footnote-23)^ | No |

1. Week 1 laboratory results seen by ID [↑](#footnote-ref-1)
2. Antimicrobial error at discharge [↑](#footnote-ref-2)
3. 30-day hospital re-admission [↑](#footnote-ref-3)
4. ED visits within 60 days of discharge related to infection [↑](#footnote-ref-4)
5. Unfavourable outcomes which include antimicrobial adverse events, catheter complications, relapse of infection or Clostridium difficile infection [↑](#footnote-ref-5)
6. Odds ratio was calculated where possible, otherwise absolute percentages used in cases where 0% and 100% results were achieved. [↑](#footnote-ref-6)
7. Serious or adverse effects [↑](#footnote-ref-7)
8. 30-day hospital re-admission [↑](#footnote-ref-8)
9. Return to hospital or clinic within 90 days for UTI [↑](#footnote-ref-9)
10. Comparing adverse effects of ertapenem (nausea/vomiting, bilateral extremity heaviness, facial swelling, cramping, hallucination, rash/petechiae and intestinal cramping and loss of appetite vs gentamicin (ototoxicity or nephrotoxicity) [↑](#footnote-ref-10)
11. Odds ratio was calculated where possible, otherwise absolute percentages used in cases where 0% and 100% results were achieved. [↑](#footnote-ref-11)
12. Odds ratio was calculated where possible, otherwise absolute percentages used in cases where 0% and 100% results were achieved. [↑](#footnote-ref-12)
13. Unfavourable outcomes, death and treatment failure [↑](#footnote-ref-13)
14. Hospital re-admissions related to OPAT [↑](#footnote-ref-14)
15. ED visits associated with infection or OPAT [↑](#footnote-ref-15)
16. Hospital re-admissions related to OPAT [↑](#footnote-ref-16)
17. OPAT related ED visits [↑](#footnote-ref-17)
18. Catheter complications [↑](#footnote-ref-18)
19. 30-day hospital re-admissions, reasons for re-admission included PICC/midline complication, antibiotic related ADR, related to index infection, relation to secondary infection and other reasons [↑](#footnote-ref-19)
20. ED visits within 60 days of discharge [↑](#footnote-ref-20)
21. Cost per re-admission due to vancomycin monitoring [↑](#footnote-ref-21)
22. Readmissions related to OPAT during OPAT [↑](#footnote-ref-22)
23. Mortality during OPAT [↑](#footnote-ref-23)
